# Heavy Metal Pollution and Source Contributions in Agricultural Soils Developed from Karst Landform in the Southwestern Region of China

**DOI:** 10.3390/toxics10100568

**Published:** 2022-09-27

**Authors:** Yuanli Qin, Fugui Zhang, Shandong Xue, Tao Ma, Linsong Yu

**Affiliations:** 1 Institute of Geophysical and Geochemical Exploration, Chinese Academy of Geological Sciences, Langfang 065000, China; 2 Planning and Natural Resources Bureau of Pingyi County, Linyi 273300, China; 3 Shandong Institute of Geophysical and Geochemical Exploration, Jinan 250013, China

**Keywords:** soil contamination, hazardous geological body, risk assessment, southwest China, PMF

## Abstract

Heavy metal pollution of soil in agricultural areas is the most prominent environmental pollution problem in China, seriously affecting human health and food security. It has become one of the environmental problems to which all sectors of society attach great importance. Soil heavy metals in the weathering area of hazardous geological bodies in southwest China have naturally high background attributes. Therefore, ecological risk assessment and analysis of potential sources of soil heavy metals in southwest China is of great significance for soil health management, soil heavy metal pollution control and territorial spatial planning. In this study, we collected 787 soil samples (0–20 cm) in Xuanwei County in China and analyzed the concentrations of As, Cd, Cr, Cu, Hg, Ni, Pb and Zn in soils. *Igeo*, *RI*, *HI* and *CR* were used to calculate the pollution levels, ecological risks and human health risks. Additionally, the PMF model and one-way ANOVA were used to identify the potential sources and discuss the factors affecting the enrichment of heavy metals. The results showed that the mean contents of the surface soils were 1.190 (Cd), 139.4 (Cr), 96.74 (Cu), 0.081 (Hg), 56.97 (Ni), 46.66 (Pb) and 130.1 (Zn) mg/kg. All heavy metals exceeded the background values of the A layer soil in Yunnan Province. The *I_geo_* showed that Cd was the most hazardous element in the study area, followed by Cu, Cr, As, Ni and Pb. The *RI* showed that low ecological risks, moderate ecological risks, considerable ecological risks and strong ecological risks accounted for 3.81%, 55.27%, 37.74% and 3.18%, respectively, of the total samples, and Cd was the main dominant element. The *HI* values of the As element in children were greater than 1, indicating a non-carcinogenic risk, and other elements’ risks were acceptable. The *CR* values of Cr and Ni were higher than their limits (1 × 10^−4^), and both had carcinogenic risks in children and adults, as did As in children. According to the PMF model, four heavy metals sources were identified: geological sources (32%), sources from mining activities (19.38%), atmospheric deposition sources (17.57%) and agricultural sources (31.05%). Thereinto, As and Pb were mainly derived from agricultural sources, Cd and Cr were mainly associated with geological sources, Cu was largely from mining activity sources, Hg was mainly from atmospheric deposition sources and Ni and Zn were mainly from geological sources, mining activities and agricultural activities. The parent material has a significant influence on the enrichment of heavy metals in the soil, and the heavy metals are significantly enriched in the carbonate parent material and quaternary parent material. Topography also plays a role in heavy metal accumulation; Cd, Cr, Cu, Ni and Zn gradually decreased with the increase in altitude, and As and Pb increased with the increase in altitude. Mn-oxide played a crucial part in the enrichment of Cu and Zn, while SOC, K_2_O and pH had little influence on the accumulation of heavy metals.

## 1. Introduction

The term “heavy metal” is based on categorization by density or molar mass, which usually refers to metals with a density greater than 4.5 g/cm^3^ [1]. In terms of soil pollution, heavy metals usually refer to cadmium (Cd), Chromium (Cr), copper (Cu), Mercury (Hg), nickel (Ni), lead (Pb) and zinc (Zn). Moreover, arsenic (As), regarded as non-metallic, has metallic behavior and is considered specifically a metalloid [2,3]. After entering the environment or ecosystem, heavy metals can exist in a variety of chemical states or chemical forms [4,5]. What is more, due to their recalcitrance, cumulativity and hypertoxicity, heavy metals would cause soil degradation and groundwater pollution, seriously reduce crop production and pose a threat to the ecosystem and human health [6,7,8,9].

In China, nearly one-sixth of agricultural land have been polluted, and inorganic pollutions were the dominant pollutants, by which 20 million hectares are contaminated by heavy metals (MEP, 2014) [10]. Thereinto, the polluted arable land in the southwest regions of China has reached 2.195 million hectares, posing a serious threat to local agricultural products and the ecological environment. Meanwhile, the regional chemical atlas of China and the 1:200,000 stream sediment survey results also show that heavy metals in soils have a naturally high background attribute in karst landform areas of the southwest regions of China, especially the Cd element [11,12]. Heavy metals pollution has become a major problem in the southwest regions of China [13,14,15,16].

Carbonate rocks are considered to be the hazardous geological bodies that cause heavy metal pollution in agricultural soils because of the secondary enrichment of carbonate parent materials during weathering and pedogenesis [16,17,18]. Soils developed in the karst landform areas of the world are generally rich in heavy metals, such as the elements of Cd, Pb and Zn. The spatial distribution of heavy metals pollution is related to the hazardous geological bodies [16,19,20]. These areas are often referred to as heavy metals high geological background regions in which heavy metals are usually high in content, low in activity and low in ecological risks [21,22,23,24,25]. In China, the southwest regions are the most widely distributed and developed areas of karst landforms, and carbonate rocks are the dominant material basis of the karst landforms [16,26]. For thousands of years, local residents have cultivated terraced fields according to the trends of the mountains and carried out agricultural activities in the carbonate parent-material areas, and some of them, such as the Honghe Hani Rice Terraces are included in the Globally Important Agricultural Heritage System (GIAHS) [27]. With the intensification of human activities and land-use change, the soil physical and chemical property configurations have changed. As a result, a part of the heavy metals are activated. Under these conditions, crops are in a state of high stress from heavy metals, resulting in heavy metals pollution in agricultural soil, which may be the reason why heavy metals have natural high background content in soils of the southwest regions of China [28,29,30]. Therefore, it is necessary, for preventing and controlling soil heavy metals pollution, to carry out a series of systematic research projects to study the distribution, the pollution levels and the ecological risks of heavy metals in karst areas. The sources of the heavy metals are also needed.

With the research into heavy metals pollution becoming the focus of attention, many scholars have carried out some related research about the content status, distribution characteristics, pollution levels and enrichment factors of heavy metals in the southwest karst landforms of China in recent years [13,14,15,16,21,28,29,30,31,32,33,34]. However, there are few studies on the sources of agricultural heavy metals, which affects the scientific judgment about soil heavy metals pollution. Therefore, it is necessary to identify the sources of heavy metals in soils, especially in the high metal background areas of hazardous geological bodies, such as carbonate rocks in the southwest regions of China, which is one of the important contents for soil pollution prevention and control.

Source apportionment methods, for example, the emission inventory method, source modeling method and receptor model, were initially carried out for the sources of particulate matter in the atmospheric environment, and now these have gradually formed a relatively complete air pollution source analysis technology system [35,36,37]. Soil source apportionment is derived from atmospheric source analysis, but it is affected by the complex characteristic of soil heavy metal pollution, such as concealment, accumulation and regionalism [38,39]. This is different from air pollution. In recent years, many methods and models have been applied to soil source analysis, such as principal component analysis-multiple linear regression (PCA-MLR), chemical mass balance (CMB), positive matrix factorization (PMF), partial least squares (PLS) and artificial neural networks (ANNs) [40,41,42,43,44,45]. Among them, the PMF model is recommended by the USEPA; it simplifies the multiple-dimensional variables and gets a few overall elements with a covariance matrix and relation matrix [46]. Plus, this method has non-negative constraints on factor loading and factor scores in the process of solving to avoid negative values in the results of the matrix factorization, and makes the obtained source component spectrum and source contribution rate have interpretable and clear meanings [47]. In addition, PMF does not require the measurement of the source profiles and uses error estimates for each individual data point to deal more reasonably with missing and imprecise data [48].

Xuanwei is located in the east of Yunnan Province, at the junction of Yunnan and Guizhou provinces, which is the main development areas of karst landforms. So far, there are few studies, such as on the spatial distribution characteristic, regional soil pollution assessment, and ecological risk assessment, on heavy metal pollution in this area. Thus, the objectives of this study were to (1) evaluate the pollution levels, the potential ecological risks and human health risks of heavy metals in hazardous geological bodies soils, (2) identify the potential sources and their contributions of heavy metals to the hazardous geological bodies soils and (3) explore the driving factors affecting heavy metal enrichment.

## 2. Materials and Methods

### 2.1. Study Area

**Figure 1:**
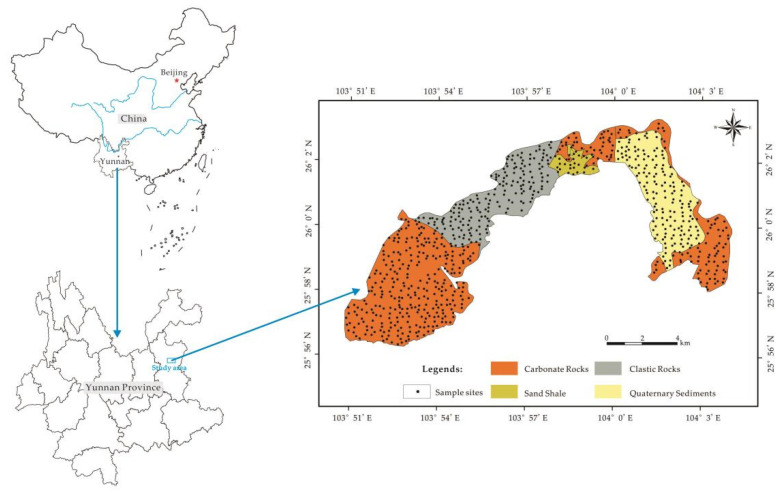
Soil lithology distribution and sampling sites of the study area.

Xuanwei County is located in Yunnan Province, China, between 25′56″~26′4″ N and 103′5″~104′4″ E (Figure 1). It covers an area of 90 km^2^. The area has a low-latitude plateau monsoon climate that is tempered by its low latitude and moderate elevation. Its average temperature is 13.4 °C and annual rainfall is 974.6 mm. The terrain in this area fluctuates greatly, with an average elevation of 2147 m. Geologically, the study area is located in the southwest margin of the Yangtze plates, which have strong geological tectonic movement and obvious folds and faults. The soil lithology in the study area is carbonate rocks, clastic rocks, sand shale and quaternary sediments (Figure 1). The soil types are latosol, alluvial soil skeleton soil and lake wetland. Compared with other regions of China, the typical farmland in this area is mainly terraced fields nestling against the mountains, and it mainly grows corn and potatoes.

### 2.2. Sample Collection and Pretreatment

In 2016, 787 samples were collected from surface soil (0–20 cm) throughout the study area according to the current situation of land use. The sampling density was 8.74 points/km^2^. The distribution of samples is shown in Figure 1. When collecting soil samples, we usually took three to five subsamples around the pre-selected sampling location and combined them into a sample by the equal quantity method. In addition, during the sampling process, debris such as root residue, gravel, stones, sand and pebbles in the soils would be removed and put them into cloth bags to take away. Furthermore, at the completion of sampling, the hand-held receiver GPS was used to locate each sampling point, and the coordinates of the sample point were recorded. The collected soils, when air-dried in a warehouse, were pounded with a rubber hammer and crushed using a mortar through a 20-mesh nylon sieve. Finally, those samples were put into plastic bottles and sent to the laboratory for testing.

### 2.3. Sample Analysis and Quality Assurance

Sample analysis and testing were completed by the Hubei Geological Research Laboratory. In the test, the content of heavy metals (As, Cd, Cr, Cu, Hg, Ni, Pb and Zn), K_2_O, Mn, SOC and soil pH values were determined. The detection methods and corresponding detection limits are shown in Table S1. Sample analysis and testing were strictly in accordance with the requirements of sample analysis quality in relevant technical standards, and soil national standard material samples were also inserted in the analysis process with the same conditions for analysis to monitor the accuracy of the analysis. The values of the allowable limits between the measured and standard values are shown in Table S1.

### 2.4. Contamination Assessment of Heavy Metals in Soils

#### 2.4.1. Geo-Accumulation Index

The geo-accumulation index (*I_geo_*) was initially used to quantitatively evaluate the degree of heavy metal pollution in sediment [49,50]. Due to its scientificity, accuracy and intuitiveness, the *I_geo_* has been widely used to evaluate soil heavy metal pollution in recent years, which was calculated as follows [49]:

(1) $$I_{geo}=Log_{2}\left[\frac{C_{i}}{k\times B_{i}}\right]$$

where *C_i_* is the concentration of heavy metal *i* in surface soil samples, while *B_i_* is the geochemical background value of element *i* in the A layer soil of Yunnan Province, and factor *k* is the correction coefficient, generally 1.5. According to Muller (1969) [49], the *I_geo_* classification is shown in Table 1.

#### 2.4.2. Ecological Risk Index

The potential ecological risk index (*RI*), which was used to evaluate the risk of heavy metals from the sedimentological perspective by integrating the toxicity levels of heavy metals, considered not only the content level but also the synergistic effect of multiple elements, the toxicity level and the environmental sensitivity [51]. The calculation formulas of the potential ecological risk index are Equations (2) and (3):

(2) $$E_{r}^{i}=T_{r}\times \frac{C_{r}^{i}}{C_{r}^{b}}$$

(3) $$RI=\sum_{i=1}^{n}E_{r}^{i}$$

where $E_{r}^{i}$ is the potential ecological hazard coefficients of element *r* at point *i*. $C_{r}^{i}$ is the measured content of element *r* at point *i*, $C_{r}^{b}$ is the soil geochemical background value of element *r*. $C_{r}$ is the toxic response factor of element *r* (i.e., As = 10, Cd = 30, Cr = 2, Cu = 5, Hg = 40, Ni = 5, Pb = 5, and Zn = 1). Its classification is shown in Table 2. RI is the comprehensive value of potential ecological hazard coefficients of heavy metals at point *i*. It is divided into four grades (Table 2).

#### 2.4.3. Health Risk Assessment

The risk assessment is the risk characterization of the adverse health effects caused by environmental pollution according to the exposure assessment model developed by the USEPA, which is generally accomplished in two steps: hazard identification and dose response assessment [52,53,54,55,56]. The equation presented in Exhibits 4–6 is used for calculating intake of children and adults by ingestion, dermal absorption, and inhalation for chemicals.

(4) $$ADD_{ingest}=\frac{CS_{i}\times IR_{ing}\times CF\times EF\times ED}{BW\times AT}$$

(5) $$ADD_{dermal}=\frac{CS_{i}\times CF\times SA\times AF\times ABS\times EF\times ED}{BW\times AT}$$

(6) $$ADD_{inhalation}=\frac{CS_{i}\times IR_{inh}\times ET\times EF\times ED}{BW\times AT}$$

where *ADD_ingestion_*, *ADD_dermal_* and *ADD_inhalation_* refer to the average daily doses through exposure pathway by ingestion, dermal absorption, and inhalation, respectively, while other parameters and theirs description and values are shown in Table 3.

The hazard quotient (*HQ*) is a function used to characterize the probability of non-carcinogenic risk. The hazard index (*HI*) is used to assess the total potential risks of noncarcinogenic effects from multiple elements. The potential carcinogenic risks (*CR*) of developing a tumor from exposure to heavy metals were calculated using the following Equations (7)–(9):

(7) $$HQ=\frac{ADD}{RfD}$$

(8) $$HI=\sum_{1}^{n}HQ_{i}$$

(9) $$CR=ADD_{i}\times SF$$

where *RfD* is the chronic reference dose, and SF is the slope factor (Table 4).

### 2.5. Positive Matrix Factorization Analysis (PMF)

PMF, as a receptor model, is an effective multi-factor data analysis method proposed by Paatero and Tapper in 1993 that is a mathematical approach for quantifying the contributions of sources to samples based on the composition or fingerprints of the sources [57,58,59]. It decomposes a matrix of speciated sample data into two submatrices: factor contributions (*G*) and factor profiles (*F*).

(10) $$X_{ij}=\sum_{k=1}^{n}G_{ik}F_{kj}+e_{ij}$$

where *X_ij_* is the concentration of element *i* in sample *j*; *G_ik_* is the concentration of element *i* in source *k*; *F_kj_* is the contribution of source *k* to sample *j*; and *e_ij_* is the residual matrix.

(11) $$Q=\sum_{i=1}^{n}\sum_{j=1}^{m}\frac{e_{ij}{}^{2}}{u_{ij}}$$

(12) uij=56×MDL

(13) $$u_{ij}=\sqrt{{\left(\delta \times c\right)}^{2}+MDL^{2}}.$$

where *u_ij_* is the uncertainty of element *i* in sample *j*, which is calculated according to method detection limit (*MDL*). When the concentration of elements is less than or equal to the corresponding *MDL*, *u_ij_* is Equation (12), and while it is larger than *MDL*, the uncertainty is (13). δ is the relative deviation, generally 5% [60,61].

### 2.6. Data Analysis and Statistics

All the data were preliminarily processed in Excel 2015 (Microsoft Corporation, Redmond, WA, USA) and SPSS 19.0 (IBM CORP, Armonk, NY, USA) software to calculate the geochemical parameters. In addition, the figures are completed by ArcGIS 10.0 (Environmental Systems Research Institute Inc., Chicago, IL, USA) and CorelDRAW X4 (Corel Corporation, Ottawa, Canada) software.

## 3. Results and Discussion

### 3.1. The Overview of Soil HMs and Geochemical Indices in the Study Area

The concentrations of chemical components in the soil are shown in Table 5. The mean content of As, Cd, Cr, Cu, Hg, Ni, Pb and Zn were 29.56, 1.190, 139.4, 96.74, 0.081, 56.97, 46.66 and 130.1 mg/kg, respectively. They were 1.61, 5.46, 2.14, 2.22, 1.40, 1.34, 1.15, 1.45 times the background values of soil in Yunnan Province [62]. Furthermore, compared with the national soil geochemical reference value, the heavy metals were 1.96 (As), 5.48 (Cd), 1.80 (Cr), 2.12 (Cu), 1.25 (Hg), 1.74 (Ni), 1.71 (Pb) and 1.48 (Zn) times higher than the corresponding high background reference value (75%) [63]. In general, the content of heavy metals obviously exceeded their background values and showed significant enrichment characteristics. The variation coefficients of heavy metals in the study area were all over 20%, specifically 58.77% (As), 52.78% (Cd), 48.50% (Cr), 137.64% (Cu), 45.95% (Hg), 34.86% (Ni), 26.40% (Pb) and 33.85% (Zn), respectively, showing high spatial variation [64].

The average content of Mn in the study area was 655.9 mg/kg, with a range of 42.44~2260 mg/kg, which was similar to its background value [62]. The coefficient of variation was 56.9%, presenting high variation. K_2_O content ranged from 0.239~4.487%, with an average of 1.468%. The variation coefficient of K_2_O was in the middle (61.22%), showing high variation. SOC was abundant in the study area, and the content ranged from 0.216~4.93%, with an average content of 1.961%. The coefficient of variation was 31.35%, indicating moderate variation. The pH in the study area varied from 4.26 to 8.30, with a median value of 5.38, and 89.71% of the sampling sites were in acidic or weakly acidic environments.

The background value content of heavy metals in southwest China was high, particularly the Cd element [65,66]. Compared with other research reported in southwest China (Table 6), the contents of Cd, Cr and Ni in the study area were similar to those reported in other geological high background areas. However, the elements such as As, Cd and Zn were lower than those in the agricultural soils of Xiangjiang River in Hunan Province [67]. The As element was similar to that of the agricultural soils in Guangdong and Almyros (GR) [6,42], but it was lower than in the soils around the lead–zinc mine [21]. Compared with the study in Table 6, the content of Hg was low compared with that in Hainan Province, which was much lower than that in the typical volcanic soils in Maicheng County of Hainan Province (0.081 vs. 49.09 mg/kg) [8]. There was little difference in the content of Cu, Pb and Zn between this study area and southwest China. Compared with Qatar, Almyros (GR) and Fogang County in southeast China, Zhuxi County in central China and Chengmai County in south China, the content of Cu and Pb were significantly different, which were 3.78, 2.78, 7.96, 1.94 and 2.89 times Cu, and 2.56, 4.76, 0.90, 1.78 and 2.40 times Pb, and 1.41, 4.36, 2.31, 0.73 and 1.94 times Zn [8,42,50,54,68].

### 3.2. Assessment of Heavy Metals Pollution

#### 3.2.1. Geo-Accumulation Index Assessment

In this paper, the high background values of soil geochemical baseline values of China were used to calculate the geo-accumulation index. The statistical results showed that the cumulative parameters of heavy metals in the study area are Cd (1.86) > Cu (0.83) > Cr (0.36) > As (0.27) > Ni (0.21) > Pb (0.10) > Zn (−0.03) > Hg (−0.15). In addition to Hg and Zn, other elements were polluted, especially Cd, which is the most polluting element with the highest value. Other elements were uncontaminated or up to a moderately polluted level.

#### 3.2.2. Ecological Risk Assessment

The potential ecological risk indexes (*RI*) showed that the samples with low ecological risks, moderate ecological risks, considerable ecological risks and strongly ecological risks accounted for 3.81%, 55.27%, 37.74% and 3.18% of the total samples, respectively. The order of contributions of the eight heavy metals to *RI* was Cd > Hg > As > Cu > Pb > Ni > Cr > Zn, and theirs contribution rates were 73.5%, 14%, 3.83%, 3.31%, 1.91%, 1.84%, 1.18% and 0.43%, respectively. Thus, it could be seen that Cd was the most hazardous element in the study area.

#### 3.2.3. Health Risk Assessment

It was an essential precondition for soil environmental management to evaluate the harm of soil heavy metals pollution to human health. An assessment of health risks of heavy metals in soil was conducted using the exposure assessment model developed by the USEPA [52,53]. Based on the exposure assessment model established by the USEPA, the average daily doses (ADD), the hazard index (HI) and the potential carcinogenic (CR) of children and adults exposed to contaminated soil through ingestion, dermal absorption and inhalation were calculated. The statistical results are shown in Table 7.

As can be seen from Table 7, the average daily doses (ADD) by ingestion were the highest, accounting for over 99.29% of the total ADD, which was the most important way of intake, while dermal and inhalation exposures were relatively low. Moreover, the ADD of children is significantly higher than that of adults, about 7.04 times higher than that of adults, indicating that the impact of heavy metal pollution on children is greater than that of adults. These results were consistent with previous studies [69,70].

The values of HI were used to quantify non-carcinogenic risks, and if the value of HI is greater than 1, that indicated a non-carcinogenic risk in the area. Table 7 shows that only the HI values of the As element in children were greater than 1, indicating a non-carcinogenic risk, while adults had no non-carcinogenic risks. In addition, although the HI value of Cr for children (0.72) was less than 1, it was also high, and it is necessary to pay attention to its hazards. Adimalla et al. (2019) reported that non-carcinogenic risks for As and Cr in children were seven times higher than for adults in India, and children were facing more acute health risks than adults, which was in line with the present study [71]. As for CR, it should be taken seriously because the CR values of the As element in children and Cr and Ni in both children and adults were higher their limits (1 × 10^−4^) [52], indicating that there were carcinogenic risks. These conclusions correspond with the results of the studies by Yang et al. (2022) about the geological high background area of heavy metals in Hainan [8].

### 3.3. Source Apportionment of Heavy Metals

**Figure 2:**
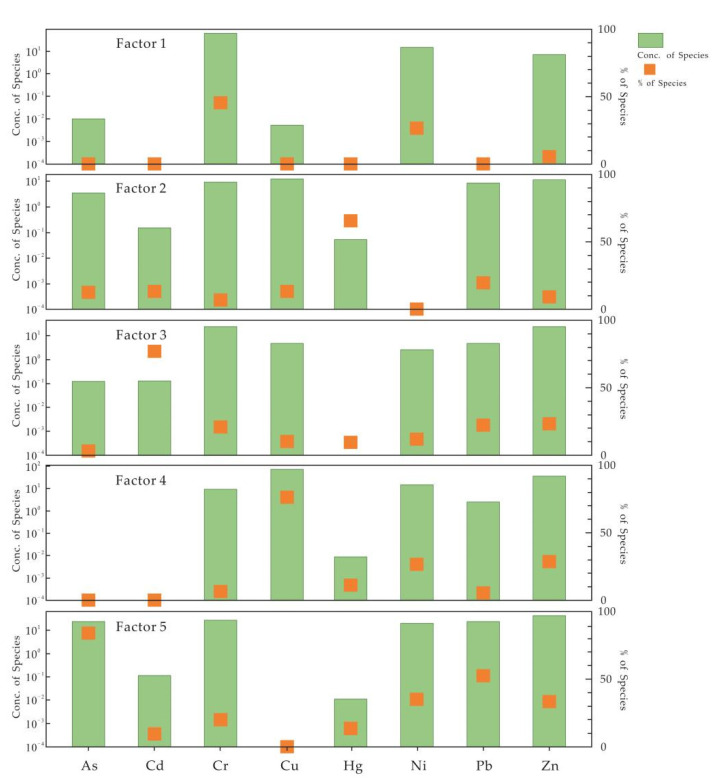
Source profiles and source contributions of soil heavy metals.

**Figure 3:**
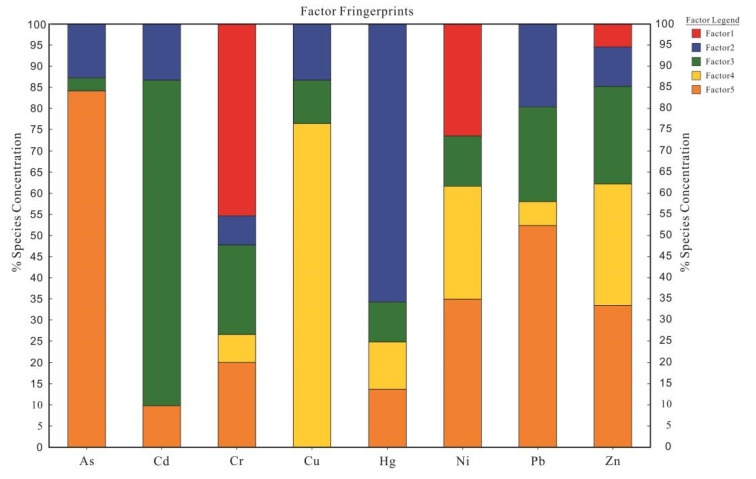
Factor profiles of heavy metal sources.

In this paper, EPA PMF 5.0 was used to identify and quantify heavy metal pollution sources. The signal-to-noise ratio (S/N) of the experimental data was greater than 10. To ensure the best prediction, 2~6 factors were respectively set for 20 iterations. Finally, Q (Robust) and Q (True) were determined to be the closest and the best effects when the number of factors were 5. Hence, eight heavy metals may have five potential sources. The source profiles and contributions are shown in Figure 2 and factor profiles are shown in Figure 3. The total contribution rates of the different sources are shown in Figure S1.

Factor 1 (which represents 9.68% of the total contributions) was characterized by the relatively high contributions of Cr and Ni, which were 45.7% and 21.8%, respectively. The CV values of Cr and Ni were 42.57% and 32.91%, indicating that they have high spatial heterogeneity. Previous studies have shown that although the content of Cr and Ni in carbonate rocks was lower than that in other parent materials, the degree of enrichment in soils formed by the weathering of carbonate rocks was higher [72]. It can be determined from Table 8 that the content of Cr and Ni in the carbonate parent materials and quaternary parent materials in the study area were significantly higher than those in other parent materials, indicating that the sources of Cr and Ni were closely related to the parent material. Zhang et al. (2020) studied Reshui Town in Xuanwei City and found that the Cr and Ni in agricultural soils mainly existed in a residual form (Cr was 81.35% and Ni was 89.03%) [72]. Therefore, factor 1 was the geological source.

The main contributing elements of factor 2, which account for the 17.57% of the total contributions, were Hg, contributing 64.6%, and Pb (20.4%), Cd (14.2%), As (13.6%), Cu (13.0%) and Zn (9.7%), which contributed the next highest. Since As is the only heavy metal existing in the atmosphere in the form of a gas phase, the migration of mercury was controlled by climate and altitude effects [73,74]. The study area was located on the Yunnan-Guizhou Plateau with an average altitude of over 2000 m and low atmospheric temperature. Under these special conditions, Hg would migrate and settle onto the high elevations along with the atmosphere, causing the accumulation of mercury in these areas. In addition, as typical emission elements of traffic pollution, Pb, Hg and Cd were mainly affected by automobile exhaust emissions, rubber tire wear, brake wear and road surface wear [75,76]. Thus, factor 2 was the anthropogenic sources of atmospheric deposition.

Cd, Zn, Cr, Pb, Ni and Cu had higher contribution values in factor 3 (which represents 22.32% of the total contributions), among which the contribution rate of Cd was as high as 76.7%, and the contribution rates of Zn, Cr, Pb, Ni and Cu were 23.5%, 20.2%, 20.8%, 13% and 10.5%, respectively. The study area is located in the karst area where the parent materials of the soil were mainly carbonate rocks. The content of Cd in the study area (1.19 mg/kg) is much higher than its background value (0.218 mg/kg). Moreover, our previous studies in this area showed that Cd mainly existed as a residual form and as potential biological components (accounting for 61.59%) that could not be absorbed and utilized by plants [65]. In addition, prior studies had shown that the fluxes of Cd in surface soils from the carbonate substrates were determined by the dual effects of secondary enrichment and parent rock inheritance [19]. This might be the reason why the content of Cd in surface soils was significantly higher than its background value. Therefore, factor 3 was a geological source.

The main loading element in factor 4 (which represents 19.38% of the total contributions) was Cu, with a contribution rate of 76.5%. Second, Ni (27.6%) and Zn (28.9%) also contributed. One-way ANOVA results showed that the contents of Cu in different parent materials were significantly different, and the content of Cu in quaternary parent materials was much higher than that in other parent materials. The CV values of Cu in the study area were high (81.38%), indicating that the distributions of Cu in the study area were inhomogeneous, and there may be point-source pollution. The study area was located in the eastern part of Yunnan Province, where a large number of IOCG-type iron-copper deposits were distributed [77]. The parent rocks were characterized by rich iron and high copper, which easily cause the enrichment of Cu, Ni and Zn during the development of the soils [78]. Therefore, factor 4 was a mining activity source.

The main contributing elements of factor 5 (which represents 31.05% of the total contributions) were As, Pb, Ni, Zn, Cr and Cd, which contributed 83.6%, 50.2%, 37.7%, 34%, 20.5% and 9%, respectively. The As element was the dominant component of factor 5. In agricultural activities, arsenic compounds were widely used in pesticides, herbicides and insecticides, which may be the potential factors causing As accumulation in agricultural soils [79,80]. In addition, the excessive use of pesticides not only caused residual toxic pollution, but also resulted in heavy metals pollution of soil because of the composition of some pesticides containing Hg, As, Cu, Zn and other heavy metals [79,81]. Therefore, factor 5 was an agricultural source.

In summary, factor 1 and factor 3 are both geological sources, so there are mainly four sources of heavy metals in the study area, namely a geological source, a mining activity source, an atmospheric deposition source and an agricultural source, with each source accounting for 32%, 19.38%, 17.57% and 31.05%, respectively.

### 3.4. Influencing Factors of Heavy Metals Enrichment

#### 3.4.1. Influence of Parent Materials on Heavy Metals

Table 8 shows statistical information regarding the content of heavy metals in the study area. One-way ANOVA showed that, except for Cr, the differences in the content of seven heavy metals in the parent materials were significant (*p* < 0.05). In soil overlying sand shale, Cd, Cu, Ni, Pb and Zn were lower than the background values of Yunnan Province, and the contents of As, Cd and Cr were lower than the average values of the study area, which indicate that the heavy metals were depleted, while the soils overlying carbonate rocks such as limestone formations were enriched with most heavy metals, such as Cd, Cu, Hg, Ni and Zn. This might be because in the pedogenesis of carbonate rocks, insoluble substances, such as iron and manganese oxides and clay minerals, remain in place, and the resolved heavy metals were constantly absorbed by them, resulting in accumulation [82]. Soil overlying clastic rocks was enriched with As, indicating that clastic rocks were one of the enrichment sources of As.

#### 3.4.2. Effects of Topographic Factors on Heavy Metals

Elevation and slope might be among the main factors affecting heavy metal enrichment in the southwest regions of China [83]. Therefore, we analyzed the one-way ANOVA in this paper (Table 8). The results showed that the content of heavy metals showed different accumulation patterns, and there were significant differences in accumulation of heavy metals at various elevations. The content of Cd, Cr, Cu, Ni and Zn increased with the decrease in elevation, and increased by 52%, 43%, 232%, 39% and 11% in the areas below 2100 m compared with those above 2250 m. What is more, the contents of As and Pb in highland areas were significantly higher than at lower-altitude spots.

#### 3.4.3. Impacts of Soil Chemical on Heavy Metals

**Figure 4:**
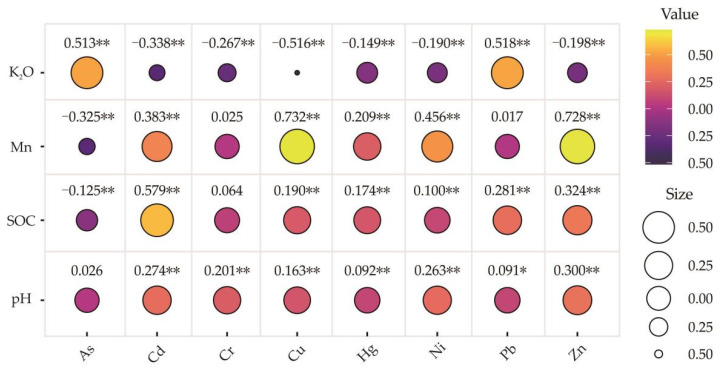
Pearson correlation matrix between soil SOC, pH, K_2_O, Mn-oxide and heavy metals in surface soils (*: *p* < 0.05; **: *p* < 0.01).

In this paper, the influence of K and Mn oxides, SOC and pH on heavy metal accumulation was discussed by calculating the Pearson correlation coefficient (Figure 4).

Iron and manganese oxides in soils have high adsorption capacity for heavy metals and are important carriers of heavy metal migration and enrichment [84,85]. In this study, a significant positive correlation was found between Mn-oxides and the heavy metals Cu and Zn (*p* < 0.01), and the concentrations of Cu and Zn increased with increasing Mn-oxides, suggesting that Mn-oxides may be the contributing factor to Cu and Zn enrichment. SOC not only affects the accumulation of heavy metals in soils but can also form a complex with heavy metal elements, affecting the migration and transformation of various forms of heavy metals [86]. There was a moderate correlation between SOC and Cd in the study area, and the correlation between SOC and other heavy metals was weak, which implied that SOC had little cumulative effect on heavy metals. This may be due to the fragile ecological environment in southwest China, serious soil degradation under natural conditions, thin reservoir of organic matter and limited adsorption capacity of soil heavy metals. Zhang Fugui et al. (2022) found the same result when they conducted a soil survey in the Hezhang area, Guizhou Province [66].

Except for the slight correlation between As and Pb and K_2_O in soils, the correlation between others and K_2_O in soil was not obvious, while the correlation between pH and all heavy metals was weak. The same research results were also found in the study of a karst area in southwest China [12,66]. As alkaline metal ions K^+^, Na^+^, Ca^2+^ and Mg^2+^ in soil solutions had the chemical properties of neutralizing H^+^ and preventing soil acidification. The study area was located in the karst area of southwest China. The widely distributed carbonate rocks provide sufficient K^+^, Na^+^, Ca^2+^, Mg^2+^ and other basal ions during weathering and soil formation to provide a buffer for soil acidification, which may be the reason why heavy metals were less affected by pH and K_2_O.

The main reason for heavy metals enrichment in the study area was the weathering and pedogenesis of soil-forming parent materials in the karst area. The enrichment of Cd, Cr and Ni was mainly from the release of soil-forming parent materials (i.e., secondary enrichment of carbonate rocks during soil formation). That was also the most important heavy metal element causing heavy metal pollution (Cd) and harm to human health (Cr and Ni) in the study area. Therefore, special attention should be paid to Cd, Cr and Ni elements in high background areas, and the monitoring network should be established to dynamically monitor the contents of Cd, Cr and Ni in soil and crop seeds. In addition, if it is not necessary, people should not come into contact with the developing soils in karst areas, especially children. In addition, As and Pb pollution caused by agricultural activities was also an important cause of harm to human health. In particular, the element As was a major contributor to both carcinogenic risks and non-carcinogenic risks. We should take some measures to limit the use of chemicals containing heavy metals in agriculture to prevent heavy metal pollution from the use of fertilizers and pesticides in agriculture. Heavy metals from mining activities and atmospheric deposition, such as Cu, Zn and Hg, were less harmful to soil and humans, and their risks were manageable.

## 4. Conclusions

In this paper, we took the heavy metals high background area in the southwest of China as the research area. By studying the concentration, pollution degrees, ecological hazards and sources of heavy metals in the soil overlying hazardous geological bodies we found that:

(1) The concentrations of As, Cd, Cr, Cu, Hg, Ni, Pb and Zn in soils of the study area were significantly enriched. Moreover, their distributions were inhomogeneous, and there might be point sources of pollution.
(2) The *I_geo_* and *RI* indexes showed that Cd was the most hazardous element in the study area. The results of a human health risk assessment showed that only the As element had non-carcinogenic risks for adults and other element risks were acceptable, whereas the carcinogenic risks in the study area were more serious. Cr and Ni had carcinogenic risks in both children and adults, and As had carcinogenic risks in children.
(3) Traceability analysis by PMF found four heavy metal pollution sources, namely geological sources (factor 1 and factor 3), atmospheric deposition sources (factor 2), sources from mining activities (factor 4) and agricultural sources (factor 5).
(4) In different parent-material areas, the enrichment characteristic of heavy metals was different except the distribution of Cr, which was weakly affected by parent materials; Cd, Cu, Hg, Ni, Pb and Zn were enriched in the parent-materials area of the carbonate zone, while As was enriched in the clastic rocks area; almost all heavy metals were depleted in the shale area and enriched in the quaternary, but their enrichment degrees were weaker than that in the carbonate area.
(5) The results of the one-way ANOVA showed that topographic factors play an essential role in the accumulation of heavy metals in soils. The content of Cd, Cr, Cu, Ni and Zn gradually decreased with the increase in altitude, and the decreased amplitude was similar in different altitude intervals. The content of As and Pb increased with the increase in altitude, and the contents were higher at high altitude.
(6) In the study area, Mn-oxide was an important factor influencing the enrichment of Cu and Zn, while SOC and K_2_O had little influence on the accumulation of heavy metals. In addition, pH had no significant effect on heavy metals accumulation.

## Figures and Tables

**Table 1:** Geo-accumulation index (*I_geo_*) classification.

Class	*I_geo_* Values	Soil Pollution
0	*I_geo_* < 0	Unpolluted
1	0 ≤ *I_geo_* < 1	From unpolluted to moderately polluted
2	1 ≤ *I_geo_* < 2	Moderately contaminated
3	2 ≤ *I_geo_* < 3	From moderately to strongly polluted
4	3 ≤ *I_geo_* < 4	Strongly polluted
5	4 ≤ *I_geo_* < 5	From strongly polluted to extremely polluted
6	5 ≤ *I_geo_*	Extremely polluted

**Table 2:** Ecological risk index and ecological hazard coefficients classification.

Class	*EI* Values	Ecological Hazard	Class	*RI* Values	Ecological Risk
0	$E_{r}^{i}$ < 40	Low level	0	150 < *RI*	Low level
1	$40\;\leq \;E_{r}^{i}$ < 80	Moderate level	1	150 ≤ *RI* < 300	Moderate level
2	$80\;\leq \;E_{r}^{i}$ < 160	Considerable level	2	300 ≤ *RI* < 600	Considerable level
3	$160\;\leq \;E_{r}^{i}$ < 320	Strongly level	3	600 ≤ *RI*	Strongly level
4	$160\;\leq \;E_{r}^{i}$	Extremely level			

**Table 3:** Parameters and their detailed description for the health risk assessment [52,53].

Parameter	Description	Unit	Adult	Child
*CS_i_*	Chemical concentration in soil	mg/kg	-	-
*IR_ing_*	Ingestion rate	mg/d	100	200
*CF*	Conversion factor	kg/mg	10^−6^	10^−6^
*EF*	Exposure frequency	days/year	350	350
*ED*	Exposure duration	years	24	6
*BW*	Averaging body weight	kg	56.8	15.9
*AT*	Averaging time	days	ED × 365	ED × 365
*SA*	Skin surface area available for contact	cm^2^/event	5700	2800
*AF*	Soil to skin adherence factor	mg/cm^2^	0.2	0.2
*ABS*	Absorption Factor	unitless	0.001	0.001
*IR_inh_*	Inhalation rate	m^3^/days	14.5	7.5
*ET*	Exposure time	hours/day	24	24

**Table 4:** Reference dose (*RfD*) and slope factor (*SF*) for heavy metals [54,55,56].

Heavy Metals	*RfD* (mg/kg/d)			*SF* (kg/d/mg)		
	Ingestion	Dermal	Inhalation	Ingestion	Dermal	Inhalation
As	3.0 × 10^−4^	1.23 × 10^−4^	4.29 × 10^−6^	1.5	1.5	1.51 × 10^1^
Cd	1.0 × 10^−3^	2.5 × 10^−5^	2.86 × 10^−6^	-	-	6.3
Cr	3.0 × 10^−3^	3 × 10^−5^	-	5.01 × 10^−1^	2.0 × 10^1^	4.2 × 10^1^
Cu	4.0 × 10^−2^	1.2 × 10^−2^	-	-	-	-
Hg	3.0 × 10^−4^	2.14 × 10^−5^	-	-	-	-
Ni	2.0 × 10^−2^	5.4 × 10^−3^	9.0 × 10^−5^	1.7	4.25 × 10^1^	8.4 × 10^−1^
Pb	1.4 × 10^−3^	5.24 × 10^−4^	-	8.5 × 10^−3^	-	4.2 × 10^−2^
Zn	3.0 × 10^−1^	6.0 × 10^−2^	-	-	-	-

**Table 5:** Geochemical statistical of chemical components in topsoil samples.

Elements	n	Me ^a^	Med ^b^	Max ^c^	Min ^d^	CV ^e^	SD ^f^	Yunnan Province Background	China Background	High Background Baseline
As	787	29.56	27.48	116.5	3.386	54.65%	16.15	18.4	12.1	14
Cd	787	1.190	1.085	5.348	0.054	47.76%	0.570	0.218	0.23	0.197
Cr	787	139.4	122.4	521.0	46.96	42.57%	59.36	65.2	68.5	68
Cu	787	96.74	57.20	355.3	14.26	81.38%	78.73	43.6	27.1	27
Hg	787	0.081	0.074	0.394	0.023	41.97%	0.034	0.058	0.087	0.056
Ni	787	56.97	53.79	180.3	11.15	32.91%	18.75	42.5	29.6	31
Pb	787	46.66	47.91	118.9	14.73	27.11%	12.65	40.6	31.2	28
Zn	787	130.1	124.1	387.2	20.30	32.30%	42.02	89.7	79	84
Mn	787	655.9	594.4	2260	42.44	56.90%	373.19	626	583	-
K_2_O	787	1.468	1.174	4.487	0.239	61.22%	0.90	1.940	2.242	-
SOC	787	1.961	1.944	4.930	0.216	31.35%	0.61	2.256	-	-
pH	787	-	5.38	8.30	4.26	12.99%	0.699	5.7	6.7	-

a: mean; b: median; c: maximum; d: minimum; e: coefficient of variation; f: standard deviation. As, Cd, Cr, Cu, Hg, Ni, Pb, Zn and Mn: mg/kg; K_2_O and SOC: %; pH: unitless.

**Table 6:** Comparison of soil heavy metals content in the study area with previous studies around the world.

Location	As	Cd	Cr	Cu	Hg	Ni	Pb	Zn	Reference
This study area	29.56	1.19	139.4	96.74	0.081	56.97	46.66	130.1	-
Baoshan City, Yunnan (CHN)	93	0.269	128	48.7	0.178	57.9	45.2	114.8	[21]
Qujing City, Yunnan (CHN)	18.1	1.18	174.1	202.0	0.09	71.1	34.9	167.2	[65]
Hezhang County, Guizhou (CHN)	24.6	2.25	176.4	89.6	0.15	65.7	41.2	173.0	[66]
Fogang County, Guangdong (CHN)	5.3	0.07	27.49	12.15	0.10	10.51	51.87	56.34	[42]
Qidong County, Hunan (CHN)	105.02	10.50	100.52	62.56	0.45	-	92.70	517.20	[67]
Zhuxi County, Hubei (CHN)	14.2	2.1	78.8	49.8	0.13	58.6	26.2	178.6	[68]
Chengmai County, Hainan (CHN)	7.06	67.51	156.88	33.43	49.09	72.47	19.48	65.57	[8]
Madrid (ES)	-	0.34	26.5	22.5	-	20.9	22.8	52.8	[54]
50Qatar	27.6	0.2	85.7	25.6	-	61.9	18.2	92.4	[50]
Almyros (GR)	2.1	3.3	39.2	34.8	0.9	19.8	9.8	29.8	[6]

As, Cd, Cr, Cu, Hg, Ni, Pb and Zn: mg/kg.

**Table 7:** Statistics of health risks exposure to heavy metals under different pathways.

		ADD (Mean)			HQ (Mean)			HI (10^−2^)	CR (10^−4^)
		Ingestion (10^−4^)	Dermal (10^−6^)	Inhalation (10^−6^)	Ingestion (10^−2^)	Dermal (10^−2^)	Inhalation (10^−2^)		
As	Children	3.560	0.998	0.870	119.0	0.812	20.30	140.0	5.490
	Adults	0.499	0.569	0.471	16.60	0.462	11.00	28.10	0.828
Cd	Children	0.144	0.040	0.035	1.440	0.161	1.230	2.813	0.002
	Adults	0.020	0.023	0.019	0.201	0.092	0.663	0.956	0.001
Cr	Children	16.80	4.710	4.110	56.10	15.70	-	71.80	11.10
	Adults	2.350	2.680	2.220	7.850	8.950	-	16.80	2.650
Cu	Children	11.70	3.270	2.850	2.920	0.027	-	2.940	-
	Adults	1.630	1.860	1.540	0.408	0.016	-	0.424	-
Hg	Children	0.010	0.003	0.002	0.325	0.013	-	0.338	-
	Adults	0.001	0.002	0.001	0.046	0.007	-	0.053	-
Ni	Children	6.870	1.920	1.680	3.440	0.036	1.860	5.340	12.50
	Adults	0.962	1.100	0.908	0.481	0.020	1.010	1.51	2.110
Pb	Children	5.630	1.580	1.370	40.20	0.301	-	40.50	0.048
	Adults	0.788	0.898	0.744	5.630	0.171	-	5.800	0.007
Zn	Children	15.70	4.390	3.830	0.523	0.007	-	0.530	-
	Adults	2.200	2.500	2.070	0.073	0.004	-	0.077	-

**Table 8:** Results of one-way ANOVA for content by parent materials and elevation.

Description		n	As	Cd	Cr	Cu	Hg	Ni	Pb	Zn
Parent materials	Clastic Rocks	182	35.57 ^a^	1.05 ^bc^	137.82 ^a^	40.42 ^c^	0.070 ^b^	53.43 ^b^	40.75 ^b^	99.96 ^c^
	Carbonate Rocks	422	32.58 ^a^	1.19 ^b^	140.79 ^a^	91.93 ^b^	0.085 ^a^	57.59 ^b^	52.89 ^a^	137.27 ^b^
	Quaternary Sediments	158	16.00 ^b^	1.38 ^a^	141.62 ^a^	185.37 ^a^	0.086 ^a^	63.44 ^a^	39.15 ^b^	154.85 ^a^
	Sand Shale	25	18.96 ^b^	1.00 ^c^	127.29 ^a^	33.26 ^c^	0.061 ^b^	35.15 ^c^	34.03 ^c^	77.70 ^d^
Elevation	2050–2100 m	161	19.60 ^c^	1.41 ^a^	151.78 ^a^	162.16 ^a^	0.087 ^a^	64.54 ^a^	40.42 ^c^	151.73 ^a^
	2100–2150 m	252	25.23 ^b^	1.27 ^ab^	150.18 ^a^	111.78 ^b^	0.081 ^ab^	59.24 ^ab^	42.07 ^c^	127.03 ^b^
	2150–2200 m	272	37.39 ^a^	1.08 ^bc^	132.96 ^ab^	60.03 ^c^	0.078 ^ab^	53.42 ^bc^	51.15 ^b^	122.24 ^b^
	2200–2250 m	85	35.59 ^a^	0.95 ^c^	112.09 ^bc^	55.57 ^c^	0.074 ^b^	49.41 ^cd^	55.97 ^a^	123.54 ^b^
	2250–2300 m	17	32.48 ^a^	0.91 ^c^	104.21 ^c^	47.57 ^c^	0.087 ^a^	46.20 ^d^	55.75 ^a^	129.25 ^b^

Significant differences post-hoc comparison were indicated by the different letters (a, b, c and d).

## Data Availability

Not applicable.

## Supplementary Materials

The following supporting information can be downloaded at https://www.mdpi.com/article/10.3390/toxics10100568/s1: Figure S1: Total contribution rate of different sources calculated by PMF; Table S1: Analytical methods and detection limits.
