# Supplementary material for: Heavy Metal Pollution and Source Contributions in Agricultural Soils Developed from Karst Landform in the Southwestern Region of China
Source: Toxics. 2022 Sep 27;10(10):568. doi: 10.3390/toxics10100568 (PMC9610029; doi:10.3390/toxics10100568)
Supplement: Supplementary file 1 [file toxics-10-00568-s001.zip › toxics-1906362-supplementary.pdf]

toxics

Supplementary Materials

Heavy Metal Pollution and Source Contributions in Agricultural Soils Developed from Karst Landform in the Southwestern Region of China

Yuanli Qin, Fugui Zhang, Shandong Xue, Tao Ma and Linsong Yu

Table S1. Analytical methods and detection limits.

Indicator	Analytical Methods	Detection Limits	Unit
As	AFS[a]	0.2	mg/kg
Cd	ICP-MS[b]	0.02	mg/kg
Cr	XRF[c]	1.5	mg/kg
Cu	XRF[c]	0.1	mg/kg
Hg	CV-AFS[d]	0.0005	mg/kg
Ni	XRF[c]	0.2	mg/kg
Pb	XRF[c]	0.2	mg/kg
Zn	XRF[c]	1	mg/kg
Mn	XRF[c]	5	mg/kg
K2O	XRF	1	%
SOC	POT[e]	0.1	%
pH	ISE[f]	0.1	unitless

a: Atomic fluorescence spectrometry;
b: Inductively coupled plasma-Mass spectrometry;
c: X-ray fluorescence spectrometry;
d: Cold vapor-Atomic fluorescence spectrometry;
e: Potentiometric method;
f: Ion selective electrode.

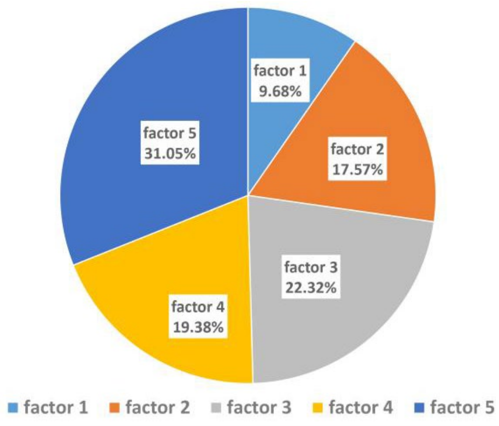

Figure S1. Total contribution rate of different sources calculated by PMF.
